# Diagnostic performance of FDG-PET/MRI and WB-DW-MRI in the evaluation of lymphoma: a prospective comparison to standard FDG-PET/CT

**DOI:** 10.1186/s12885-015-2009-z

**Published:** 2015-12-23

**Authors:** Ken Herrmann, Marcelo Queiroz, Martin W. Huellner, Felipe de Galiza Barbosa, Andreas Buck, Niklaus Schaefer, Paul Stolzman, Patrick Veit-Haibach

**Affiliations:** Department of Nuclear Medicine, University Hospital Zurich, Rämistrasse 100, CH-8091 Zurich, Switzerland; Department of Nuclear Medicine, Universitätsklinikum Würzburg, Oberdürrbacher Str. 6, DE-97080 Würzburg, Germany; Department of Neuroradiology, University Hospital Zurich, Rämistrasse 100, CH-8091 Zurich, Switzerland; Department of Diagnostic and Interventional Radiology, University Hospital Zurich, Rämistrasse 100, CH-8091 Zurich, Switzerland; Department of Medical Oncology, University Hospital Zurich, Rämistrasse 100, CH-8091 Zurich, Switzerland; University of Zurich, Zurich, Switzerland

**Keywords:** Whole-body, WB-DW-MRI, FDG, FDG-PET/CT, FDG-PET/MRI, Lymphoma

## Abstract

**Background:**

Use of FDG-PET/CT for staging and restaging of lymphoma patients is widely incorporated into current practice guidelines. Our aim was to prospectively evaluate the diagnostic performance of FDG-PET/MRI and WB-DW-MRI compared with FDG-FDG-PET/CT using a tri-modality PET/CT-MRI system.

**Methods:**

From 04/12 to 01/14, a total of 82 FDG-PET/CT examinations including an additional scientific MRI on a tri-modality setup were performed in 61 patients. FDG-PET/CT, FDG-PET/MRI, and WB-DW-MRI were independently analyzed. A lesion with a mean ADC below a threshold of 1.2 × 10^−3^ mm^2^/s was defined as positive for restricted diffusion. FDG-PET/CT and FDG-PET/MRI were evaluated for the detection of lesions corresponding to lymphoma manifestations according to the German Hodgkin Study Group. Imaging findings were validated by biopsy (*n* = 21), by follow-up imaging comprising CT, FDG-PET/CT, and/or FDG-PET/MRI (*n* = 32), or clinically (*n* = 25) (mean follow-up: 9.1 months).

**Results:**

FDG-PET/MRI and FDG-PET/CT accurately detected 188 lesions in 27 patients. Another 54 examinations in 35 patients were negative. WB-DW-MRI detected 524 lesions, of which 125 (66.5 % of the aforementioned 188 lesions) were true positive. Among the 188 lesions positive for lymphoma, FDG-PET/MRI detected all 170 instances of nodal disease and also all 18 extranodal lymphoma manifestations; by comparison, WB-DW-MRI characterized 115 (67.6 %) and 10 (55.6 %) lesions as positive for nodal and extranodal disease, respectively. FDG-PET/MRI was superior to WB-DW-MRI in detecting lymphoma manifestations in patients included for staging (113 vs. 73), for restaging (75 vs. 52), for evaluation of high- (127 vs. 81) and low-grade lymphomas (61 vs. 46), and for definition of Ann Arbor stage (WB-DW-MRI resulted in upstaging in 60 cases, including 45 patients free of disease, and downstaging in 4).

**Conclusion:**

Our results indicate that FDG-PET/CT and FDG-PET/MRI probably have a similar performance in the clinical work-up of lymphomas. The performance of WB-DW-MRI was generally inferior to that of both FDG-PET-based methods but the technique might be used in specific scenarios, e.g., in low-grade lymphomas and during surveillance.

## Background

Use of fluorodeoxyglucose positron emission tomography/computed tomography (FDG-PET/CT) for staging and restaging of lymphoma patients is now clinical routine and is widely incorporated into current practice guidelines [1]. Recent advances in magnetic resonance imaging (MRI) technology and MRI sequences have led to the introduction of whole-body diffusion-weighted MRI (WB-DW-MRI) and allowed for calculation of apparent diffusion coefficients (ADC) [2]. WB-DW-MRI is expected to improve staging accuracy due to the potential improvement in lesion-to-background contrast [3]. Previously published studies comparing WB-DW-MRI and FDG-PET/CT reported kappa values for method agreement ranging from 0.51 to 0.85 [4, 5]. The fact that all major vendors offer hybrid scanners combining MRI, PET, and/or CT technology allows for direct comparison of single imaging modalities as well as hybrid approaches [6].

Potential advantages of WB-DW-MRI in comparison with either FDG-PET/CT or FDG-PET/MRI include no radiation burden, the possibility of protocol standardization, and high tumor-to-background contrast; in addition, image acquisition times are comparable.

Promising initial results encouraged authors to advocate WB-DW-MRI as a potential replacement for FDG-PET/CT [7]. However, as yet no prospectively validated ADC criteria have been established for differentiation of lymphomatous from non-lymphomatous lymph nodes when using WB-DW-MRI. Moreover, few data are currently available regarding the performance of FDG-PET/MRI and WB-DW-MRI as compared with FDG-PET/CT in lymphoma patients.

The aim of this study was therefore to prospectively evaluate the evaluate the diagnostic performance of FDG-PET/MRI and WB-DW-MRI compared with FDG-FDG-PET/CT using a tri-modality PET/CT-MRI system that allows for a one-stop examination in a realistic everyday clinical setting including pretreatment staging, interim and end of treatment restaging, and surveillance.

## Methods

### Patient population

From April 2012 through January 2014, all patients referred for a clinical FDG-PET/CT examination for either staging or restaging lymphoma were offered an additional scientific MRI within a tri-m odality setup. A total of 82 examinations were performed in 61 patients, with 15 patients undergoing more than one scan (ten patients, two examinations; four patients, three examinations; and one patient, four examinations). No further patient inclusion criteria were applied. Exclusion criteria were unwillingness to participate in the study, claustrophobia, MRI-incompatible medical devices (e.g., cardiac pacemakers, neurostimulators, cochlear implants, and insulin pumps), or possible presence of metallic fragments in the body. This prospective study was approved by the ethics committee of the Canton of Zurich and signed informed consent was obtained from all patients prior to the examinations.

### FDG-PET/CT and MRI

Sequential FDG-PET/CT and MRI were performed on a tri-modality PET/CT-MRI setup (full ring, time-of-flight Discovery PET/CT 690, 3 T Discovery MR 750w, both GE Healthcare, Waukesha, WI, USA). The dedicated MRI- and CT-compatible shuttle transfer mechanism connecting the MRI and PET/CT systems allowed for PET/CT scanning free of radiofrequency (RF) coil-induced artifacts and ascertained the placement of dedicated RF coils for MRI without repositioning of the patient [8, 9].

Patients fasted for at least 4 h prior to injection of a standard FDG dose of 4.5 MBq per kg body weight [10]. After an uptake time of 30 min the patient was positioned on the shuttle table in the MRI suite and MRI acquisition covering the region from the head to the upper thighs was started. The images were acquired by use of a GEM whole-body suite (GE Healthcare, Waukesha, WI, USA). The MRI protocol included a T1-weighted three-dimensional spoiled gradient echo pulse sequence (LAVA) and diffusion-weighted images obtained in the axial plane, both divided into four stations, with a total MRI scan duration of 15–20 min (see Table 1 for scanning parameters).

**Table 1:** MRI scanning parameters

Parameter	T1w LAVA	DWI EPI-STIR
Repetition time/echo time (ms)	4.3/1.3	4175/100
Echo train length	NA	NA
Flip angle (°)	12	NA
Inversion time (ms)	NA	200
Parallel imaging acceleration factor	2	2
Receiver bandwidth (kHz)	142.86	250
Field of view (cm)	50	44
Matrix	288 × 224	64 × 128
b value (s/mm)	NA	0, 50, 500
NEX	1	NA
Number of directions	NA	3

Abbreviations: *T1w LAVA* T1-weighted spoiled gradient echo pulse sequence, *DWI* diffusion-weighted imaging sequence, *EPI-STIR* echo planar imaging-short time inversion recovery, *NEX* number of excitations, *NA* not applicable

After completion of the MRI, coils were removed and the patients were transferred to the PET/CT, still positioned on the shuttle board. In this way, it was ensured that positioning of the patient within the PET/CT and the MRI scanners was exactly the same.

After shuttle transfer to the adjacent PET/CT system (after an overall uptake time of 60 min), unenhanced low-dose CT and PET emission data were acquired from the mid-thigh to the vertex of the skull. The low-dose CT was acquired during shallow breathing in the head, upper thorax, and pelvis areas and with non-forced expiration breath hold in the diaphragm and upper abdomen.

Tube voltage was 120 kV (peak), reference tube current 12.35 mA/slice, automated dose modulation range 15–80 mAs/slice, collimation 64 × 0.625 mm, pitch 0.984:1, rotation time 0.5 s, field of view (FOV) 50 cm, and noise index 20 %. CT image sets were reconstructed using an iterative algorithm [Adaptive Statistical Iterative Reconstruction (ASIR), GE Healthcare].

The PET data were acquired in 3-D time of flight (TOF) mode with a scan duration of 2 min per bed position, a 23 % overlap of bed positions, an axial FOV of 153 mm, and a 700-mm-diameter FOV. The emission data were corrected for attenuation by use of the low-dose CT and iteratively reconstructed [matrix size 256 × 256, VUE Point FX (3D TOF-OSEM) with 3 iterations, 18 subsets]. Images were filtered in image space using an in-plane Gaussian convolution kernel with a full-width at half-maximum (FWHM) of 4.0 mm, followed by a standard axial filter with a three-slice kernel. This procedure has been used in this standard way in other studies as well [11].

### Image processing

The acquired FDG-PET/CT and MRI images were transmitted to a dedicated review workstation (Advantage Workstation, Version 4.5, GE Healthcare, Milwaukee, WI, USA) that enables review of the PET, CT, and MRI images side by side or in fused/overlay mode (FDG-PET/CT; FDG-PET/MRI). Due to use of the calibrated three-modality system, no software-based image registration was necessary. A previously conducted study validated the accuracy of image registration, with less than 4 mm lateral misalignment between CT, PET, and MRI data sets, which is similar to the intrinsic error assessed with phantom measurements [12].

### Image analysis

Analysis was performed by a board-certified nuclear medicine physician and a board-certified radiologist with substantial experience in FDG-PET/CT. All images were evaluated for the presence of lymphoma manifestations according to the German Hodgkin Study Group (GHSG) protocol guidelines, including a total of 34 possible anatomic sites divided into nodal or organ involvement [3, 13–15].

Nodal involvement was considered to comprise lymphoma manifestation at any of the following sites: Waldeyer’s ring, upper cervical, cervical, supraclavicular, infraclavicular, axillary, lung hilum, iliac and inguinal (right or left), upper mediastinum, lower mediastinum, liver hilum, spleen, splenic hilum, celiac, mesenteric, and para-aortic. Organ involvement was characterized as presence of a positive lesion for lymphoma in the lung (right or left), liver, pleura, skeleton, pericardium, bone marrow, or any other organ not previously described.

With regard to WB-DW-MRI, a positive lymphoma manifestation was represented by a high-signal lesion on high b-value WB-DW-MRI and a low signal on the corresponding ADC map, using a mean ADC of 1.2 × 10^−3^ mm^2^/s as the threshold.

For assessment of lymphoma manifestation on FDG-PET/CT and FDG-PET/MRI, a combination of morphologic and functional findings was used. The morphologic criteria for lymphoma manifestation were presence of a mass-like lesion, presence of enlarged lymph nodes greater than 1.0 cm in the short axis (and 1.5 cm for angular lymph nodes), cluster formation, irregular boundary of the lymph node capsule, and extracapsular lymph node spread. The functional criterion was defined as presence of an FDG-positive lesion with higher focal FDG uptake than liver activity (Deauville criteria, see below). For FDG-negative lesions, the morphologic criteria were used.

### Image validation and follow-up

Imaging findings were validated by biopsy (*n* = 21), by follow-up imaging comprising CT, FDG-PET/CT, and/or FDG-PET/MRI (*n* = 32), or by clinical follow-up (*n* = 25). Due to loss to follow-up, five examinations (all negative on FDG-PET/CT) could not be further validated. Verification by biopsy was only available for one lesion per patient; however, FDG-PET/CT was then used as the reference method for comparison of the other modalities. The positivity of FDG-PET/CT and FDG-PET/MRI was based on Deauville criteria and lesions with FDG uptake higher than the liver uptake were considered positive (Deauville scores 4 and 5) [16]. The median follow-up estimated by the inverse Kaplan-Meier method was 9.1 months (range 0.0–21.3 months, median 8.7 months).

### Statistical analysis

All statistical tests were performed using SPSS Statistics Version 22 (IBM, Armonk, NY, USA). Quantitative values were expressed as mean ± standard deviation or median and range as appropriate. Comparisons of means and related metric measurements were performed using Student’s t-test and the Wilcoxon signed rank test, respectively. All statistical tests were conducted two-sided and a p value less than 0.05 was considered to indicate statistical significance.

## Results

### Patient characteristics

Sixty-two patients with a mean age of 55 ± 20 years (median 62; range 20–90) were prospectively included in this study. A total of 82 examinations were performed for primary staging (*n* = 14) and restaging (*n* = 68). Restaging consisted of interim examinations during ongoing therapy (*n* = 14), examination after end of treatment (*n* = 19), and surveillance (*n* = 35).

The majority of the examinations were done for assessment of Hodgkin’s disease (*n* = 28) or diffuse large B-cell lymphoma (*n* = 26) (for details, see Table 2). One patient who presented with suspicion for lymphoma was found to have sarcoidosis upon histologic verification and was later excluded, leaving 61 patients for lymphoma analysis.

**Table 2:** Patient characteristics

Lymphoma entity	Examinations	Patients
Hodgkin’s disease	28	21
DLBCL	26	18
CLL	2	2
Follicular lymphoma	5	5
MALT	1	1
Mantle cell lymphoma	11	6
Marginal cell lymphoma	2	2
Peripheral T-cell lymphoma	2	2
Peripheral B-cell lymphoma	1	1
Large cell lymphoma	1	1
Angioblastic lymphoma	2	1
Sarcoidosis (excluded)	1	1
Mycosis fungoides	1	1
Total included	82	61

Abbreviations: *DLBCL* diffuse large B-cell lymphoma, *CLL* chronic lymphocytic leukemia, *MALT* mucosa-associated lymphoid tissue

### Detectability rate

Overall, 188 lesions were considered positive in 29 examinations in 27 patients (see Table 3). Another 53 examinations in 34 patients were considered negative for lymphoma.

**Table 3:** Clinical consensus in respect of Ann Arbor stage

	Stage I	Stage II	Stage III	Stage IV
All	6	8	8	7
Primary staging	3	2	6	2
Interim scan	1	1	1	1
End of Tx	1	2	0	1
Surveillance	1	3	1	3

FDG-PET/MRI accurately detected 188 lesions, yielding a sensitivity of 100 % compared with FDG-PET/CT. On the other hand, WB-DW-MRI detected 524 lesions, of which 125 (66.5 % of 188) lesions were true positive and 319 false positive findings. WB-DW-MRI accordingly missed 63 true positive (33.5 % of 188) lesions.

### Detection of nodal vs. extranodal disease

Of the 188 lesions positive for lymphoma, 170 represented nodal disease while 18 were found in extranodal sites. The distribution of FDG-positive lymphoma manifestations according to localization is shown in Table 4.

**Table 4:** Lymphoma manifestations according to the German Hodgkin Study Group (GHSG) protocol guidelines (*n* = 188)

Region	N
Waldeyer left	3
Waldeyer right	4
Upper cervical left	9
Upper cervical right	9
Cervical left	11
Cervical right	10
Supraclavicular left	11
Supraclavicular right	10
Infraclavicular left	9
Infraclavicular right	7
Axillary left	7
Axillary right	8
Upper mediastinum	12
Lower mediastinum	10
Lung hilum left	2
Lung hilum right	5
Spleen	6
Splenic hilum	1
Liver hilum	4
Celiac	4
Para-aortic	6
Mesenteric	6
Iliac left	4
Iliac right	9
Inguinal left	3
Inguinal right	8
Organ	10

**Fig. 1:**
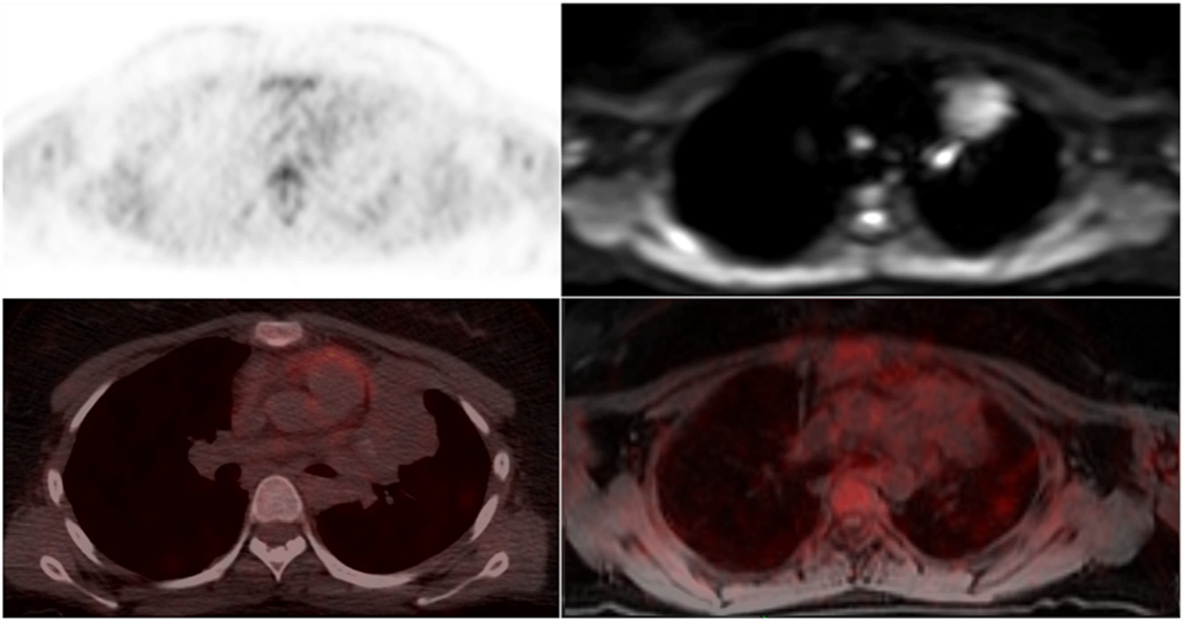
A male patient with Hodgkin’s disease stage IIIE. PET/CT/MRI after two cycles of chemotherapy. Top: Axial PET shows very faint uptake in the anterior mediastinal lesion; axial WB-DW-MRI (b value = 800) shows restricted diffusion (calculated ADC_mean_ = 0.96 × 10^−3^ mm^2^/s). Bottom: FDG-PET/CT and FDG-PET/MRI show a residual mediastinal mass without significant FDG activity. FDG-PET/CT and FDG-PET/MRI after the end of treatment confirmed complete response

**Fig. 2:**
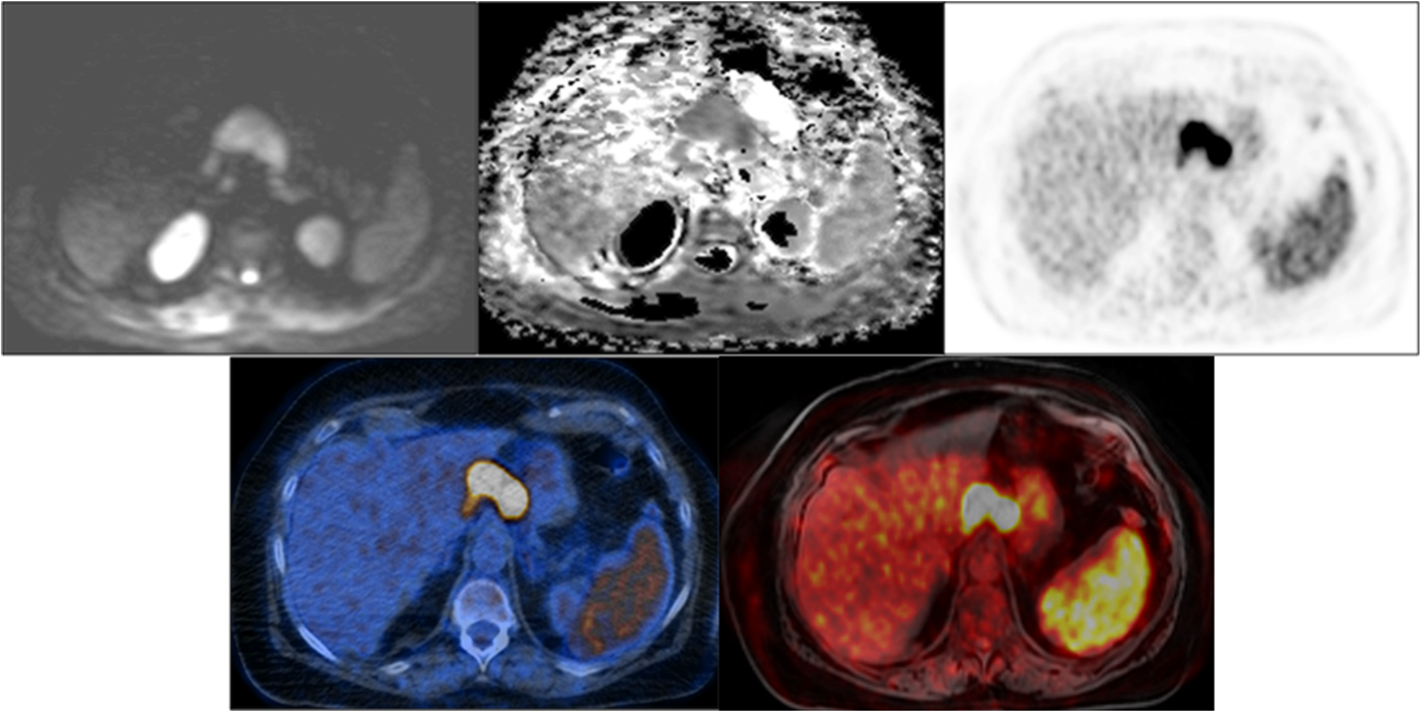
A female patient with a diffuse large B-cell lymphoma stage IVB. PET/CT/MRI for initial staging. Top: Axial WB-DW-MRI (b value = 800) and axial ADC map show restricted diffusion in a lymph node conglomerate in the upper abdomen (calculated ADC_mean_ = 0.72 × 10^−3^ mm^2^/s), but no restricted diffusion in the spleen (calculated ADC_mean_ = 1.37 × 10^−3^ mm^2^/s). Axial PET shows uptake in the same lymph node conglomerate but also diffuse uptake in the spleen, which was significantly higher than liver uptake. Bottom: FDG-PET/CT and FDG-PET/MRI show FDG avidity in both the lymph node mass and the spleen, indicating lymphoma manifestation

FDG-PET/MRI detected all 170 instances of nodal disease and also identified all 18 extranodal lymphoma manifestations; by comparison, WB-DW-MRI characterized 115 (67.6 %) and 10 (55.6 %) lesions as positive for nodal and extranodal disease, respectively (Fig. 1). Among the extranodal manifestations, splenic involvement was the source of the greatest discrepancy, with WB-DW-MRI detecting only 50 % of cases and yielding false positive findings in three other patients (Fig. 2).

### Staging vs. restaging

Among the 188 lesions positive for lymphoma, 113 (60.1 %) were found in patients included for primary staging and 75 (39.9 %) in those included for restaging. Among the primary staging patients, FDG-PET/MRI accurately detected all positive lesions while WB-DW-MRI identified 73 (64.6 %) lesions. Among the patients undergoing restaging, FDG-PET/MRI and WB-DW-MRI characterized 75 (100 %) and 52 (69.3 %) lesions, respectively.

### Interim vs. end of treatment vs. surveillance

FDG-PET/CT and FDG-PET/MRI detected the same number of lesions in patients who underwent examination during ongoing therapy (*n* = 16), after the end of treatment (*n* = 12), and during surveillance (*n* = 47), while WB-DW-MRI detected nine (56.3 %), six (50.0 %), and 37 (78.7 %) lesions, respectively.

### Hodgkin’s disease (HD) vs. diffuse large B-cell lymphoma (DLBCL) and low- and intermediate- vs. high-grade lymphoma

Of the 82 examinations included, 28 were indicated for HD and 26 for DLBCL, accounting for a total number of 66 and 61 of the detected lesions, respectively. WB-DW-MRI accurately detected 40 lesions (60.6 %) in HD patients and 41 DLBC patients (67.2 %). Fifty-four examinations were performed for evaluation of high-grade lymphomas, with FDG-PET/MRI detecting 127 positive lesions and WB-DW-MRI, 81 (63.8 %). The remaining 28 examinations were performed for evaluation of low- and intermediate-grade lymphomas. Here, all 61 lesions considered positive for lymphoma were accurately detected by FDG-PET/MRI, while 46 (75.4 %) were detected with WB-DW-MRI.

### Ann Arbor stage

In 18 examinations, WB-DW-MRI and FDG-PET/MRI agreed with respect to Ann Arbor stage (8 stage 0, 1 stage I, 0 stage II, 4 stage III, 1 stage IIIS, and 4 stage IV). Among the other 64 examinations, WB-DW-MRI resulted in upstaging in 60 cases, including 45 patients who were free of disease as determined by FDG-PET/CT (WB-DW-MRI changed the stage from 0 to I in 9 patients, 0 to II in 10 patients, 0 to III in 25 patients, and 0 to IV in 1 patient), and downstaging in four (from IIIS to III in 1 patient and from IV to III in 3 patients). Among the 27 patients with positive findings for lymphoma, WB-DW-MRI and FDG-PET/MRI agreed in ten patients (34.5 %) while upstaging was observed in 15 (51.7 %) and downstaging in four (13.8 %).

A summary of the comparative results for FDG-PET/CT, FDG-PET/MRI, and WB-DW-MRI is provided in Table 5.

**Table 5:** Comparison of number of positive lymphoma lesions detected by FDG-PET/CT, FDG-PET/MRI, and WB-DW-MRI

	PET/CT = PET/MRI	WB-DW-MRI	Detectability rate with WB-DW-MRI	*P* value
Lesions detected	188	125	66.5 %	<0.001
Nodal disease	170	115	67.6 %	<0.001
Extranodal disease	18	10	55.6 %	0.004
Staging	113	73	64.6 %	0.008
Restaging	75	52	69.3 %	0.010
Interim	16	9	56.3 %	0.169
End of treatment	12	6	50.0 %	0.083
Surveillance	47	37	78.7 %	0.058
High-grade	127	81	63.8 %	0.003
Low-grade	61	46	75.4 %	0.032
HD	66	40	60.6 %	0.006
DLBCL	61	41	67.2 %	0.009

Abbreviations: *HD* Hodgkin’s disease, *DLBCL* diffuse large B-cell lymphoma

## Discussion

The results of this study show that the diagnostic performance of FDG-PET/MRI in lymphoma patients in a realistic everyday clinical setting is equal to that of FDG-PET/CT, which is nowadays widely accepted as the modality of choice for staging and restaging of lymphoma patients. On the other hand, the performance of WB-DW-MRI seems to be inferior to that of FDG-PET/CT/MRI in various respects, most notably for staging, differentiation of nodal and extranodal disease, and differentiation of high-grade and low-grade lymphoma. The performance of WB-DW-MRI was better for evaluation during surveillance and in the assessment of low-grade lymphomas (cf. Table 5).

FDG-PET/CT and FDG-PET/MRI showed agreement for all lesions, which is not too surprising given that the PET component was the same. Differences between MRI and CT have been especially described for detection of bone marrow (MRI superior) [17] and lung involvement [3] (CT superior). However, in our study population, in which lung involvement was present in only three patients and bone marrow involvement in only one patient, FDG-PET/CT and FDG-PET/MRI were in agreement due to the increased FDG uptake in all corresponding lesions.

The equivalent performance of FDG-PET/CT and FDG-PET/MRI in patients with lymphoma was recently confirmed in a retrospective study including 33 patients and a total of 702 lymph node stations [18]. Using FDG-PET/CT as the reference standard, FDG-PET/MRI had a sensitivity of 93.8 % and a specificity of 99.4 %, results which are in line with those of our study.

For WB-DW-MRI, ADC values were determined for any lesions visually detectable, as no definite cut-offs have previously been reported. We evaluated the entire data set using different cut-offs for the ADC (data not shown), and the cut-off selected (mean ADC threshold of 1.2 × 10–3 mm2/s) performed best in terms of overall accuracy. Nevertheless, the difficulty in identification of a cut-off explains the very high number of false positive lesions on WB-DW-MRI, which in general detected two-thirds of lymphoma lesions. Difficulty in deriving an optimal cut-off for the ADC value was also reported by Punwani et al. in 39 patients undergoing WB-DW-MRI and FDG-PET/CT before and after two cycles of chemotherapy. Interim ADC values in patients with adequate FDG-PET/CT response were not statistically different from those in patients without an adequate response [7]. PET imaging detects lymphoma activity on the basis of tumor glucose metabolism, while WB-DW-MRI does so on the basis of the motion of water molecules in a densely cellular environment. Our findings show – as do those of several previous publications – that tumor cellularity as detected by WB-DW-MRI may not be an adequate marker for lymphoma activity to the same extent as glycolytic metabolism. This inference is supported by the findings of Wu et al., who concluded, on the basis of results in patients with histologically proven DLBCL, that SUV for PET and ADC for WB-DW-MRI are different indices for the characterization of lymphomas [19].

When our patient population was categorized into different subsets according to lymphoma manifestation (nodal vs. extranodal disease), indication (staging vs. restaging), timepoint of examination (during ongoing therapy, after end of treatment, and surveillance) and grade of lymphoma (low- vs. high-grade and HD vs. DLBCL), WB-DW-MRI failed to achieve the lesion detectability offered by FDG-PET/CT or FDG-PET/MRI in any of the subsets. Kwee et al. recently reported that WB-DW-MRI did not provide any advantage over MRI without DWI in 108 newly diagnosed lymphoma patients [3]. Slightly improved results were reported by Tsuji et al., who compared FDG-PET/CT and MRI in 28 malignant lymphoma patients prior to any treatment and after two cycles of chemotherapy [20]. While concordant findings were reported in 22/28 (79 %) patients, significant differences were nevertheless found between FDG-PET/CT and MRI. The best results were obtained in the study of Mayerhoefer and co-workers, who reported WB-DW-MRI to have a region-based sensitivity of 97 % compared with FDG-PET/CT in known FDG-avid lymphoma histologic subtypes [21].

In our study, the results of WB-DW-MRI were not statistically different from those of the reference standard, FDG-PET/CT, with respect to interim/end of treatment imaging and surveillance. These findings are to an extent similar to the results of Mayerhoefer and co-workers in lymphomas with variable FDG avidity [21]. However, the impact of the low numbers of lesions in the relevant subsets in our study has to be borne in mind.

As WB-DW-MRI achieved an almost comparable detection rate to FDG-PET/CT among patients undergoing surveillance, this method might be considered for follow-up of this subgroup of patients when baseline imaging with WB-DW-MRI is available, especially given that FDG-PET/CT in general is not recommended for this purpose [1].

WB-DW-MRI showed inferior results in the evaluation of extranodal disease and for overall restaging. Only a few studies have evaluated the accuracy of WB-DW-MRI and FDG-PET/MRI for detection of extranodal disease. Results of other studies have suggested that, overall, WB-DW-MRI and FDG-PET/MRI may have an advantage compared with FDG-PET/CT for this purpose [22, 23], especially when considering bone marrow involvement. In our study, only one patient presented bone marrow infiltration, so we cannot offer further comment on this aspect. However, we did find that diffuse splenic involvement may not be reliably detected by WB-DW-MRI; this confirms previous observations by Toledano-Massiah and colleagues [22] and reflects the fact that restricted diffusion may be observed even in a normal spleen.

For therapy response assessment, FDG-PET/MRI has proved to be feasible and reliable [24]. Most studies describe an elevation in the ADC mean value as suggestive of response to treatment [25–27]. Our study has shown that, when used for restaging, WB-DW-MRI performed less well than FDG-PET/MRI in detecting lymphoma activity. This finding suggests that the use of WB-DW-MRI to assess treatment response of lymphoma may underestimate the true number of lesions and that careful evaluation is required in order to avoid false negative findings.

We observed only moderate agreement between WB-DW-MRI and FDG-PET/MRI or FDG-PET/CT concerning determination of the Ann Arbor stage. One of the reasons for this may be the lack of standardized criteria for definition of lymphoma involvement on WB-DW-MRI, which may be considered responsible for the very high number of false positive lesions in our study. As indicated above, we tested our results with different ADC thresholds (data not shown). When the threshold was changed, however, the values for sensitivity and specificity altered in opposite directions (e.g., sensitivity increased but specificity decreased) and no improvement in overall accuracy was achieved. Another drawback is that no parameters have been defined for the evaluation of extranodal disease. Hence, the reproducibility of WB-DW-MRI is limited and may also be partly dependent on the MRI scanner used.

Overall, our results indicate that the similarity in diagnostic performance of FDG-PET/CT and FDG-PET/MRI reported previously in various solid tumors also holds true for FDG-avid lymphoma types. While the findings of most studies cited above are generally in line with our own results, the reported inferiority of WB-DW-MRI compared with FDG-PET-based techniques is somewhat at odds with a few other studies in the literature. One potential explanation is our choice of everyday setting including a wide range of histologies and different clinical situations ranging from staging to surveillance, as well as the use of a tri-modality system with MRI being performed separately from the PET component. Moreover, it is well known that bone marrow, spleen, and lymph nodes retain high signal intensity and are therefore difficult to assess with WB-DW-MRI [28].

When interpreting the results of this study, several limitations have to be taken into account. First, histopathology as the reference standard of choice was not available in all lesions (ethically this was not possible), though it was usually available in patients referred for initial staging. However, FDG-PET/CT is widely accepted as a reference standard to determine disease in lymphoma [1]. Additionally, our study did not define a threshold for lesion size in WB-DW-MRI and consequently, we detected a very high number of false positive lesions, resulting in overestimated upstaging. However, even without such a threshold, WB-DW-MRI was unable to detect all of the lesions that were positive on FDG-PET. Finally, we used a tri-modality PET/CT-MRI setup rather than simultaneous PET//MRI and thus the attenuation correction for the PET component was always based on the low-dose CT.

## Conclusion

In summary, our results indicate that FDG-PET/CT and FDG-PET/MRI have a similar performance in the clinical work-up of lymphomas, while WB-DW-MRI is inferior to both FDG-PET-based methods. WB-DW-MRI can, however, be used in specific scenarios, e.g., in low-grade lymphomas as well as imaging during surveillance.

## Abbreviations

WB-DW-MRI: Whole-body diffusion-weighted magnetic resonance imaging
MRI: Magnetic resonance imaging
PET: Positron emission tomography
FDG: Fluorodeoxyglucose
CT: Computed tomography
ADC: Apparent diffusion coefficient
CT: Computed tomography
RF: Radiofrequency
MBq: Megabecquerel
GEM: Geometry embracing method
FOV: Field of view
GHSG: German Hodgkin Study Group
DLBCL: Diffuse large B-cell lymphoma
CLL: Chronic lymphocytic leukemia
MALT: Mucosa-associated lymphoid tissue
